# CRISPR-Cas9 Editing Induces Loss of Heterozygosity in the Pathogenic Yeast Candida parapsilosis

**DOI:** 10.1128/msphere.00393-22

**Published:** 2022-11-23

**Authors:** Lisa Lombardi, Sean A. Bergin, Adam Ryan, Evelyn Zuniga-Soto, Geraldine Butler

**Affiliations:** a School of Biomolecular and Biomedical Science, Conway Institute, University College Dublin, Belfield, Dublin, Ireland; University of Georgia

**Keywords:** CRISPR-Cas9, *Candida parapsilosis*, loss of heterozygosity, genome editing

## Abstract

Genetic manipulation is often used to study gene function. However, unplanned genome changes (including single nucleotide polymorphisms [SNPs], aneuploidy, and loss of heterozygosity [LOH]) can affect the phenotypic traits of the engineered strains. Here, we compared the effect of classical deletion methods (replacing target alleles with selectable markers by homologous recombination) with CRISPR-Cas9 editing in the diploid human-pathogenic yeast Candida parapsilosis. We sequenced the genomes of 9 isolates that were modified using classic recombination methods and 12 that were edited using CRISPR-Cas9. As a control, the genomes of eight isolates that were transformed with a Cas9-expressing plasmid in the absence of a guide RNA were also sequenced. Following gene manipulation using classic homologous recombination, only one strain exhibited extensive LOH near the targeted gene (8.9 kb), whereas another contained multiple LOH events not associated with the intended modification. In contrast, large regions of LOH (up to >1,100 kb) were observed in most CRISPR-Cas9-edited strains. LOH most commonly occurred adjacent to the Cas9 cut site and extended to the telomere in four isolates. In two isolates, we observed LOH on chromosomes that were not targeted by CRISPR-Cas9. Among the CRISPR-edited isolates, two exhibited cysteine and methionine auxotrophy caused by LOH at a heterozygous site in *MET10*, approximately 11 and 157 kb downstream from the Cas9 target site, respectively. C. parapsilosis isolates have relatively low levels of heterozygosity. However, our results show that mutation complementation to confirm observed phenotypes is required when using CRISPR-Cas9.

**IMPORTANCE** CRISPR-Cas9 has greatly streamlined gene editing and is now the gold standard and first choice for genetic engineering. However, we show that in diploid species, extra care should be taken in confirming the cause of any phenotypic changes observed. We show that the Cas9-induced double-strand break is often associated with loss of heterozygosity in the asexual diploid human fungal pathogen Candida parapsilosis. This can result in deleterious heterozygous variants (e.g., stop gain in one allele) becoming homozygous, resulting in unplanned phenotypic changes. Our results stress the importance of mutation complementation even when using CRISPR-Cas9.

## INTRODUCTION

The opportunistic fungal pathogen Candida parapsilosis is a member of the CUG-Ser1 clade (1) and the second or third most frequently isolated *Candida* species, depending on geographical region and patient cohort (2). Unlike Candida albicans, C. parapsilosis has been associated with nosocomial infection outbreaks worldwide (3–6). Severe C. parapsilosis infections are a concern in intensive care units and neonatal intensive care units, where they are associated with neonatal mortality (7, 8).

Genetic manipulation of the diploid genome of C. parapsilosis is fundamental for studying the pathobiology of this species, which often cannot be directly extrapolated from C. albicans (2, 9). Site-directed mutagenesis in C. parapsilosis has been accomplished by homologous recombination using the *SAT1* flipper cassette containing a dominant selection marker (reviewed in reference 10) or nutritional selection markers to replace the gene of interest in an auxotrophic background (9). More recently, CRISPR-Cas9 technology was adapted for the C. parapsilosis *sensu lato* complex (C. parapsilosis, Candida orthopsilosis, and Candida metapsilosis), which allows markerless editing in prototrophic strains to introduce homozygous or heterozygous mutations, delete genes, and tag proteins (11–18). We are currently using the plasmid-based CRISPR-Cas9 system, pCP-tRNA (12), to systematically disrupt genes in C. parapsilosis CLIB214.

Despite being a common approach for studying gene function, the construction of mutant strains can be associated with unplanned genome rearrangements, including aneuploidy and loss of heterozygosity (LOH) (19–25). Aneuploidy entails gain or loss of a chromosome or a chromosome segment, resulting in a change in gene dosage. LOH results in loss of genetic information from one of the two chromosome homologs as a consequence of either chromosome loss (monosomy) or replacement of information on one homologous chromosome with a sequence from the other. In Candida albicans, rates of both aneuploidy and LOH increase in response to stress (26, 27). Aneuploidy and LOH are also frequently found in strains of C. albicans that underwent manipulation in the laboratory (19–25). In fact, two commonly used transformation methods (lithium acetate and electroporation) promote changes in chromosome copy number, possibly by increasing chromosome nondisjunction, and exposure to heat preferentially triggers aneuploidy or LOH, depending on the length and intensity of the exposure (22). It is now known that deleting *ura3* in the C. albicans reference strain SC5314 to generate CAI-4 resulted in trisomy of Chr2 and/or Chr3 (19, 20), and additional manipulations generating double (*ura3 his1*) or triple (*ura3 his1 arg4*) auxotrophic strains resulted in further changes (20). Large tracts of LOH (up to 1,330 kb) were also found on multiple chromosomes in other SC5314-derived strains (25).

Both aneuploidy and LOH can affect the phenotypic traits of a strain, which may be erroneously linked to the genome change that was intentionally introduced (28, 29). For example, in the process of deleting serine aspartic protease genes in C. albicans, a *sap4 sap5 sap6* triple mutant was constructed in which LOH coincidentally resulted in the loss of the *SAP2-2* allele, present as a heterozygous locus (*SAP2-1/SAP2-2*) on chromosome R. The inability of the resulting strain to use proteins as a sole nitrogen source was initially assumed to be due to the absence of Saps4-6 but was actually caused by the loss of the *SAP2-2* allele (23). Similarly, spontaneous LOH of the right arm of chromosome 3 determined sensitivity to the DNA-damaging agent methyl methane sulfonate (MMS), depending on which *MBP1* allele was retained (24).

Although CRISPR-Cas9 revolutionized the landscape of gene editing, an increasing number of studies show that this technology can also induce aneuploidy and LOH in eukaryotic cells (30–33). A recent study found that CRISPR-Cas9-generated C. albicans strains contain numerous unwanted genomic changes, albeit to a lesser extent than the strains generated with other methods (31). To evaluate the extent of LOH and aneuploidy induced by gene editing in C. parapsilosis, we sequenced the genomes of 9 strains that were manipulated by replacing each allele with nutritional markers (*CdHIS1/CmLEU2*) by homologous recombination (9) and 12 CRISPR-Cas9 genetically modified strains (10 edited strains, 1 gene deletion, and 1 strain that was transformed but did not contain the desired edit). We also included eight strains transformed with a Cas9-expressing plasmid in the absence of the guide RNA as a control. We showed that transformation is associated with aneuploidy in Candida parapsilosis because it was observed even in the control strains that were transformed with a plasmid and were not genetically modified. Moreover, we found that LOH occurs using both CRISPR and CRISPR-free methods, but it is dramatically more common and extensive in CRISPR-Cas9-edited strains, particularly on chromosomes targeted by Cas9.

We also show that unplanned LOH on chromosome 8 can result in cysteine and methionine auxotrophy. Overall, our study stresses the importance of confirming a direct relationship between genotype and phenotype when editing genes in diploid species, particularly when using CRISPR-Cas9.

## RESULTS

### Homologous recombination is associated with unplanned genome changes in C. parapsilosis.

**FIG 1:**
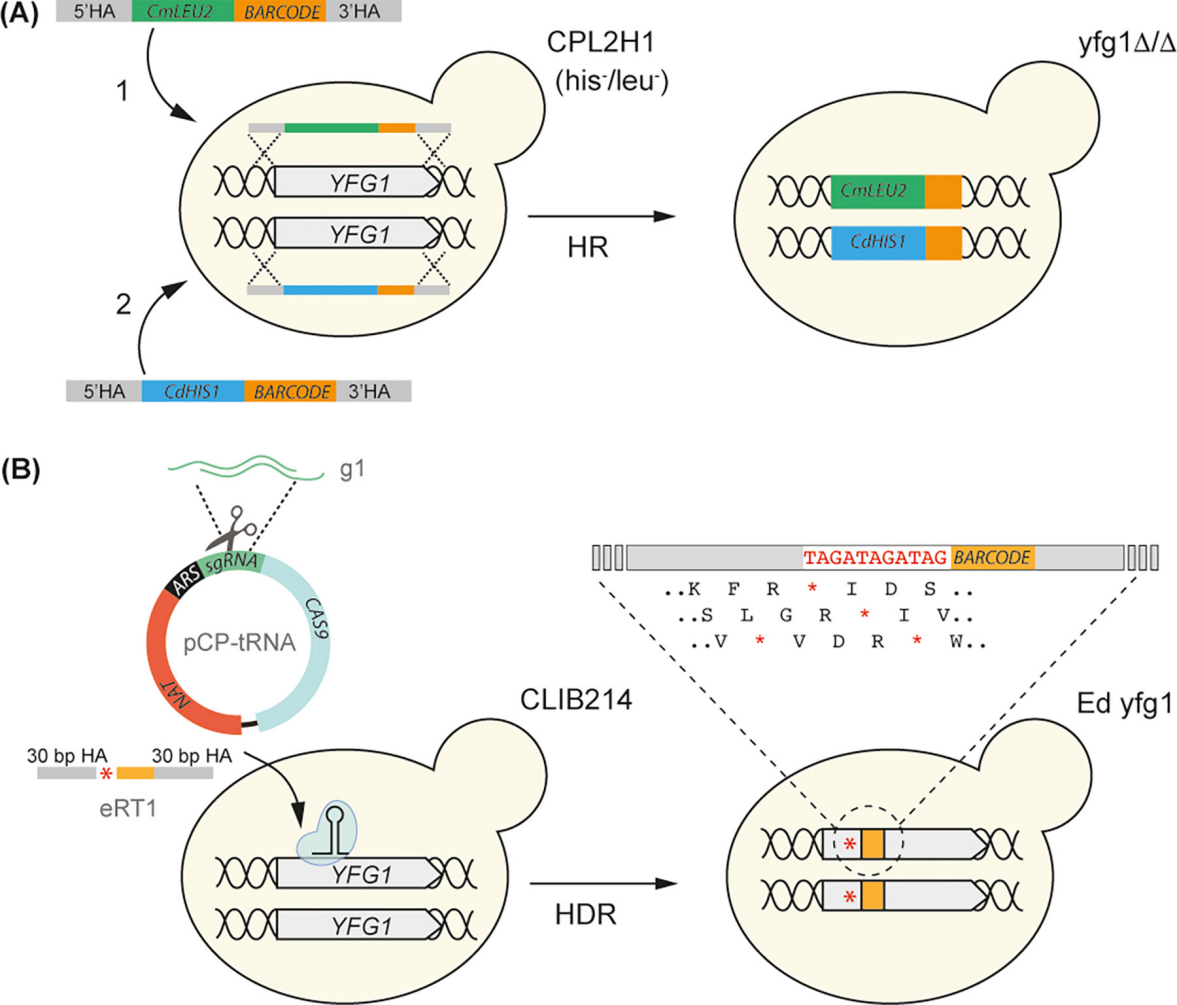
Deletion by homologous recombination (A) and CRISPR-Cas9 editing (B) in C. parapsilosis. (A) Deletion strains were constructed in C. parapsilosis CPL2H1, a *his1 leu2* derivative of C. parapsilosis CLIB214 by homologous recombination (HR) with nutritional markers (9). Deletion of the gene of interest (your favorite gene [*YFG*]) was obtained by replacing one allele with *LEU2* from Candida maltosa (first transformation, 1) and the second allele with *HIS1* from Candida dubliniensis (second transformation, 2). A barcode was also included in each deletion strain. 5′/3′HA, 5′/3′ homology arm. (B) For CRISPR-Cas9 editing, guides were designed to target Cas9 cleavage to the first third of each *YFG*. The repair templates (e.g., eRT1) contain 11 bp with stop codons in each of the 3 possible reading frames (red asterisk) and a unique barcode (in orange), flanked by two short homology arms (HAs) to drive homology-directed repair (HDR). The short double-stranded (dsDNA) guides (e.g., g1) were cloned into the pCP-tRNA plasmid, which harbors *CAS9*, the autonomously replicating sequence (*ARS*), and a nourseothricin marker cassette (*NAT*) (12). Simultaneous transformation of the pCP-tRNA (e.g., pCP-tRNA1) targeting a gene (e.g., *YFG1*) and the corresponding RT (e.g., eRT1) results in the insertion of premature stop codons (red asterisk) in both alleles by HDR (e.g., Ed yfg1).

**FIG 2:**
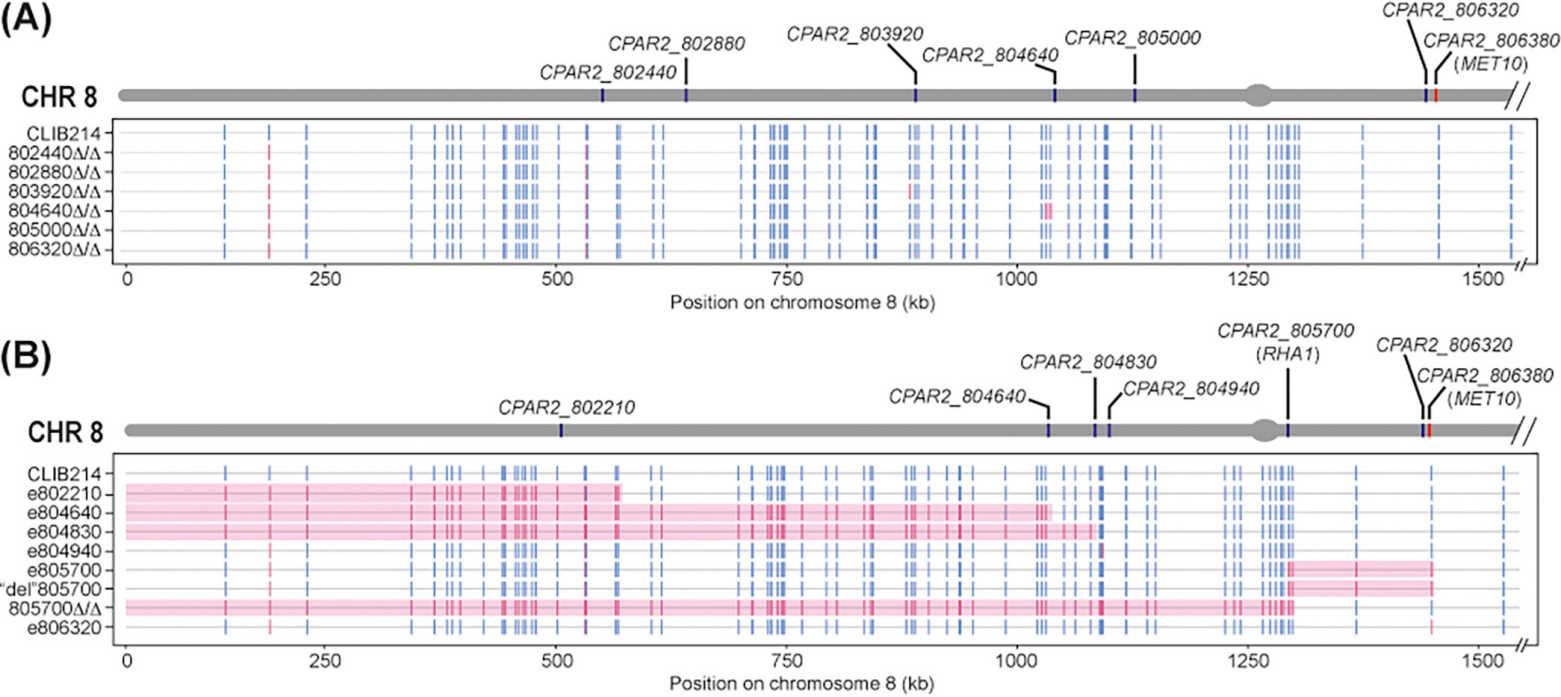
Homologous recombination and CRISPR-Cas9 induce LOH on chromosome 8 but to different extents. Heterozygous and homozygous sites (in blue and pink, respectively) on chromosome 8 in CLIB214 and strains modified using homologous recombination with nutritional markers (A) or CRISPR-Cas9 (B), with respect to the reference genome (CDC317). The gray bar on top represents chromosome 8, with target genes in blue and *MET10* in red. The centromere is indicated by the gray oval. LOH tracts (defined as LOH at a minimum of two adjacent heterozygous SNPs) are shaded in pink. The final 593 kb of the chromosome is not shown because there are no informative variants in this region (downstream position, +1,525,083) (Table S1). (A) LOH is visible in 804640Δ/Δ; the remaining strains that were targeted on chromosome 8 either do not show LOH, or they do on a different chromosome (Table 1). CLIB214, C. parapsilosis CLIB214; 802440Δ/Δ, both alleles of *CPAR2_802440* were deleted following homologous recombination with *CdHIS1* and *CmLEU2* (and so on for the other strains and genes). (B) Loss of heterozygosity at one site in e806320 results in a premature stop codon in both alleles of *CPAR2_806380* (*MET10*). The same heterozygous variant is lost as a result of LOH in strains e805700 and del805700. CLIB214, C. parapsilosis CLIB214; e802210, stop codons inserted in *CPAR2_802210* (and so on for the other strains and genes); “del”805700, a strain in which Cas9 likely cut at *CPAR2_805700* but the repair template was not incorporated; 805700Δ/Δ, both alleles of *CPAR2_805700* were deleted using CRISPR-Cas9.

We previously described the construction of homozygous deletions of 73 transcription factors and 16 protein kinases in C. parapsilosis, generated by using homologous recombination to replace both alleles at a single locus with either *CdHIS1* or *CmLEU2* (9) (Fig. 1A). To assess the effect of transformation and/or homologous recombination on the rest of the genome, we sequenced the genomes of nine homozygous null mutants that were generated using this method. C. parapsilosis is, on average, less heterozygous than C. albicans (0.1 to 0.4 SNPs/kb versus 3 SNPs/kb, respectively) (34, 35). To maximize the chances of detecting LOH on the same chromosome as the targeted gene, we selected strains in which the target was followed by regions containing at least some heterozygous sites. Many of these are on chromosome 8; as a consequence, strains edited in genes located on chromosome 8 were overrepresented in the sequenced strains (Tables 1 and 2, Fig. 2A, and see Table S1 in the supplemental material). LOH was defined as loss of heterozygosity at a minimum of two adjacent heterozygous sites.

10.1128/msphere.00393-22.3 TABLE S1 Table of genotypes for C. parapsilosis CLIB214 and strains modified using traditional homologous recombination or CRISPR-Cas9 or transformed with the pCP-tRNA expressing Cas9 but not containing the guide RNA. Download Table S1, XLSX file, 0.05 MB. Copyright © 2022 Lombardi et al. 2022 Lombardi et al. https://creativecommons.org/licenses/by/4.0/ This content is distributed under the terms of the Creative Commons Attribution 4.0 International license.

The deletion strains were constructed in a C. parapsilosis CLIB214-derived *his1 leu2* auxotrophic background (CPL2H1) (9) (Fig. 1A). The names of the deleted strains (e.g., 301940Δ/Δ) indicate the target gene (e.g., *CPAR2_301940*). All the isolates contained the planned modifications; however, all of them also shared a region of LOH extending over 267 kb on chromosome 1 (represented by loss of two heterozygous sites; Table S1), suggesting that this event happened when the CPL2H1 parental strain was constructed (9) (Table 1). Seven strains did not contain any additional LOH. In one strain (804640Δ/Δ), two heterozygous sites were lost within 8.9 kb of the target gene (Fig. 2, Table 1, and Table S1). In addition, we observed two regions of LOH ranging in size from 1.4 to >5.6 kb on chromosomes 2 and 5 in isolate 203540Δ/Δ, and the shared LOH on chromosome 1 is extended to 1,280 kb, caused by loss of an additional heterozygous site (Table 1; Table S1). Strain 804640Δ/Δ (cla4Δ/Δ [9]) also has four copies of chromosome 5 (Fig. S1).

**TABLE 1:** LOH in CPL2H1-derived homozygous deletion mutants obtained by homologous recombination with *CdHIS1/CmLEU2*^*e*^

Strain no.	Strain	Start of target gene	LOH coordinates^*a*^	LOH range (kb)^*a*^
1	302230Δ/Δ	Chr3: 981,411	None	
2	302310Δ/Δ	Chr3: 999,958	None	
3	802440Δ/Δ	Chr8: 549,537	None	
4	804640Δ/Δ^*b*^	Chr8: 1,034,238	Chr8: 1,025,374 to 1,029,742	8.9
5	203540Δ/Δ	Chr2: 712,580	Chr2: 1,113,080 to 1,114,531	1.4^*c*^
5	203540Δ/Δ	Chr2: 712,580	Chr5: 862,893 to 868,523	5.6
5	203540Δ/Δ	Chr2: 712,580	Chr1: 529,693 to 1,542,886	1,013^*d*^
6	802880Δ/Δ	Chr8: 641,406	None	
7	805000Δ/Δ^*b*^	Chr8: 1,122,896	None	
8	803920Δ/Δ	Chr8: 884,849	None	
9	806320Δ/Δ^*b*^	Chr8: 1,436,518	None	
1–9		Shared LOH	Chr1: 1,542,886 to 1,810,008	267

a LOH coordinates identify the positions of the first and last variants in the LOH block. The LOH range is defined as the distance from the ATG to the most distant homozygous site. When LOH is observed on nontargeted chromosomes, the LOH range is defined as the distance between the first and the last variant in the LOH block.

b Described in reference 9 as cla4Δ/Δ, wor1Δ/Δ, and kis1Δ/Δ deletions, respectively.

c Seven heterozygous variants separate the deleted gene from the LOH located 401 kb downstream, so the LOH is not considered near the target gene.

d This LOH tract extends from the LOH on the telomere of chromosome 1 that is shared among all the isolates.

e The strains were obtained as described in reference 9. Gray color highlights LOH occurring near the target gene.

10.1128/msphere.00393-22.1 FIG S1 Aneuploidy in C. parapsilosis CLIB214-derived strains modified using CRISPR-Cas9 technology (A and B), CRISPR-free gene deletion (C), or transformed with the empty pCP-tRNA plasmid (D and E). Aneuploidy in strains, evidenced by log_2_ of mean coverage (log_2_ MC) across the genome. (A and B) Strains e205070 and e804640 have an extra copy of chromosome 6 and 5, respectively. (C) Strain 804640Δ/Δ (described in reference 9 as cla4Δ/Δ) has four copies of chromosome 5. (D and E) Both transformants 3 and 8 selected on nourseothricin after transformation with the empty pCP-tRNA plasmid have one extra copy of chromosome 7. Download FIG S1, TIF file, 1.7 MB. Copyright © 2022 Lombardi et al. 2022 Lombardi et al. https://creativecommons.org/licenses/by/4.0/ This content is distributed under the terms of the Creative Commons Attribution 4.0 International license.

### CRISPR-Cas9 induces loss of heterozygosity in C. parapsilosis.

We are currently using a plasmid-based CRISPR-Cas9 system (12) to disrupt additional genes in C. parapsilosis (Fig. 1B). We therefore sequenced the genomes of 12 strains generated by targeting 10 different genes. Edited strains (i.e., where 11 bases containing stop codons in all open reading frames and a unique barcode are introduced) are indicated using the prefix “e” (e.g., e301940; both alleles of *CPAR2_301940* are edited), and gene deletions are indicated using Δ/Δ (e.g., 805700Δ/Δ; both alleles of *CPAR2_805700* are deleted). For one gene (*CPAR2_805700*), three different isolates were sequenced, (i) e805700, in which both alleles were edited by transformation with pCP-805700 plasmid and eRT-805700 repair template (Fig. 2B; Table S5); (ii) “del”805700, in which we attempted, but failed, to delete the gene by transforming with pCP-805700 and a deletion repair template (delRT-805700) (Fig. 2B; Table S5); and (iii) 805700Δ/Δ, in which the gene was successfully deleted after transforming pCP-805700-2 and repair template delRT-805700-2 (Fig. 2B; Table S5).

10.1128/msphere.00393-22.7 TABLE S5 Plasmids and oligonucleotides used for CRISPR-Cas9 modifications. Download Table S5, DOCX file, 0.02 MB. Copyright © 2022 Lombardi et al. 2022 Lombardi et al. https://creativecommons.org/licenses/by/4.0/ This content is distributed under the terms of the Creative Commons Attribution 4.0 International license.

The designed changes were present in all strains except one (“del”805700, in which transformation with the CRISPR-Cas9 system targeting *CPAR2_805700* did not result in deletion of the gene). Two edited strains (e205070 and e804640) contained three copies of chromosomes 6 and 5, respectively (Fig. S1). We observed LOH in 9 of the 12 edited strains (Table 2, Fig. 2, and Table S1).

LOH occurred more frequently on the chromosome on which the target gene was located (7/9 strains), suggesting that it may be induced by the Cas9-induced double-strand break (DSB) (Table 2; Table S1). This is shown in detail for chromosome 8 (Fig. 2B). The extent of the LOH regions ranges from at least 6.5 kb to more than 1,100 kb, and the LOH may reach the telomere in four isolates (Table 2; Fig. 2B).

**TABLE 2:** LOH in CLIB214-derived strains modified using CRISPR-Cas9 technology^*e*^

Strain no.	Strain	Cas9 cut site	LOH coordinates^*a*^	LOH range (kb)^*a*^
1	e101740	Chr1: 386,438	Chr5: 104,538 to 661,431	557
2	e205070	Chr2: 1,028,668	Chr2: 1,022,177 to 1,024,638	6.5
3	e301780	Chr4: 417,148	None	
4	e301940	Chr3: 898,895	Chr2: 1,540,134 to 1,619,639	79
5	e802210	Chr8: 504,595	Chr8: 142,590 to 567,069	362^*b*^^,^^*c*^^,^^*d*^
6	e804640	Chr8: 1,033,704	Chr8: 142,590 to 1,041,458	899^*b*^^,^^*d*^
7	e804830	Chr8: 1,080,807	Chr8: 142,590 to 1,078,006	938^*b*^
8	e804940	Chr8: 1,097,623	None	
9	e805700	Chr8: 1,290,224	Chr8: 1,292,562 to 1,446,962	154
10	805700Δ/Δ	Chr8: 1,288,808	Chr8: 142,590 to 1,296,622	1,146^*b*^^,^^*c*^
11	e806320	Chr8: 1,436,180	None	
12	“del”805700	Chr8: 1,290,224	Chr8: 1,292,562 to 1,446,962	154

a LOH coordinates identify the positions of the first and last variants in the LOH block. The LOH range is defined as the distance from the cut site to the most distant homozygous site. If LOH is observed on nontargeted chromosomes, the LOH range is defined as the distance between the first and the last variant in the LOH block.

b Likely reaches the telomere.

c The LOH also extends on the other side of the cut site in e802210 and 805700Δ/Δ (62 kb and 8 kb, respectively). See Fig. 2B.

d In e802210 and e804640, there are two and one heterozygous variants, respectively, near the end of the LOH tract (see Table S1 in the supplemental material); this may be due to two adjacent regions of LOH or an error in basecalling.

e Gray color highlights LOH starting at or near the Cas9 cut site. All the strains contain a premature stop codon, except 805700Δ/Δ, in which the target gene was deleted.

In two CRISPR-Cas9-edited strains, we also observed LOH on chromosomes that were not targeted by Cas9 (Table 2). In contrast, no LOH was observed in the genomes of eight isolates that were transformed with pCP-tRNA, which expresses *CAS9* but does not contain a guide RNA targeting Cas9 to a specific gene. However, aneuploidy (an extra copy of chromosome 7) was observed in two isolates (Fig. S1; Table S1).

### LOH on chromosome 8 can result in cysteine and methionine auxotrophy.

**FIG 3:**
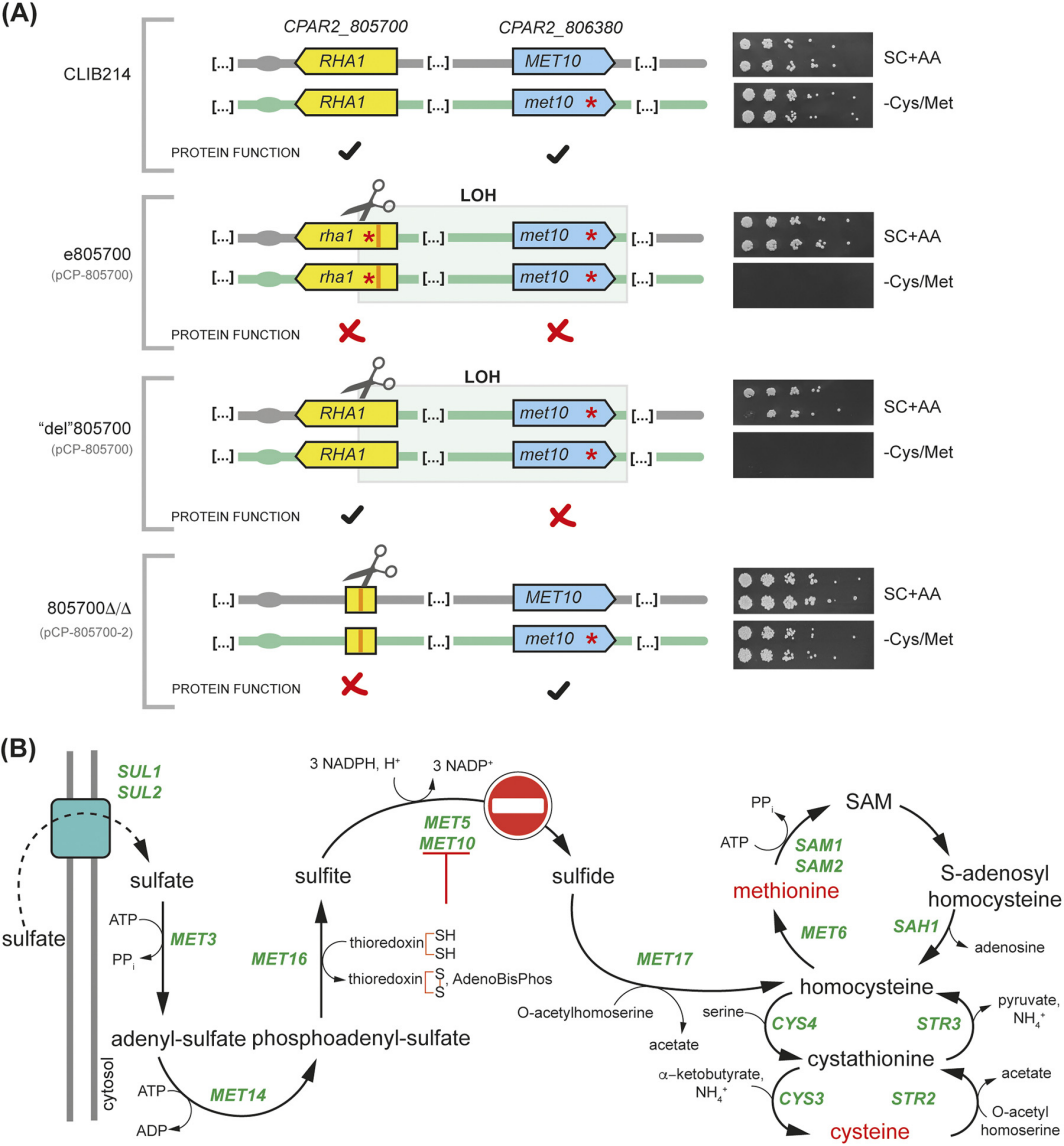
LOH-induced loss of function of the sulfite reductase Met10 causes cysteine and methionine auxotrophy. (A) The diagrams on the left show the presence of functional alleles of *RHA1* and *MET10* or of alleles containing stop codons (red asterisks). The plasmids used to target *CPAR2_805700* (*RHA1*) are indicated in gray underneath the name of each strain. Note that 805700Δ/Δ was generated using a different guide (contained in plasmid pCP-805700-2) than e805700, and “del”805700 (in which *CPAR2_805700* was targeted by the guide RNA contained in plasmid pCP-805700). Images on the right show the growth of serial dilutions of two biological replicates of each strain on SC with all amino acids or SC missing cysteine and methionine. In C. parapsilosis CLIB214, one allele of *CPAR2_806380* (*MET10*) has a premature stop codon. Strains that have undergone homozygosis at this position, generating two *MET10* alleles with stop codons, fail to grow in the absence of cysteine and methionine (e.g., e805700 and “del”805700). Strain 805700Δ/Δ contains a partial deletion of *RHA1* with no LOH at *MET10*. Cas9 target sites are indicated with a scissors icon. (B) Sulfur assimilation pathway in Saccharomyces cerevisiae (37). In the absence of cysteine and methionine, yeast cells can import sulfate from the extracellular environment and then gradually reduce it to sulfide, which is then incorporated into homocysteine. Homocysteine is then funneled into the methyl cycle to produce methionine, and into the transsulfuration pathway to synthesize cysteine. *MET10* and *MET5* encode the two catalytic subunits (α and β, respectively) of the α_2_β_2_ heterotetrametric enzyme sulfite reductase. Loss of function of Met10 shuts down the pathway, thus making cells auxotrophic for cysteine and methionine. SAM, *S*-adenosylmethionine.

While testing the phenotypes of CRISPR-Cas9 edited strains, we noticed that one isolate containing stop codons in *CPAR2_805700* (*RHA1*) on chromosome 8 failed to grow in the absence of the sulfur-containing amino acids cysteine or methionine (Fig. 3). This strain (e805700) was generated by targeting *RHA1* with the plasmid pCP-805700 and a repair template designed to introduce stop codons. *RHA1* encodes a zinc cluster transcription factor that acts as a positive filamentation regulator (36) and has never been shown to be involved in sulfur metabolism.

To further characterize the observed phenotype, we attempted to use CRISPR-Cas9 to delete *RHA1*, using plasmid pCP-805700 and a repair template designed to delete the open reading frame. The deletion strategy was unsuccessful: the resulting strains (called “del”805700) contained a functional *RHA1* gene, but they again failed to grow in the absence of cysteine or methionine (Fig. 3). Comparing the genomes of C. parapsilosis CLIB214, e805700 (edited *RHA1*), and “del”805700 showed that both CRISPR-Cas9-manipulated strains had a stretch of LOH on chromosome 8 (1,292,562 to 1,453,338) extending from *RHA1* to *CPAR2_806380* (*MET10*) (Table 2; Fig. 3). In C. parapsilosis CLIB214, one allele of *MET10* contains a premature stop codon (Table S1; Table S2). In both e805700 and “del”805700, LOH resulted in homozygosis at this site that caused loss of function of *MET10*; both alleles contain a C2600A SNP converting Ser867 into a stop codon. *MET10* encodes one of the catalytic subunits of the sulfite reductase, which reduces sulfite to sulfide in the sulfur assimilation pathway (Fig. 3B). This enzyme is a heterotetramer composed of 2 α and 2 β subunits (α_2_β_2_), encoded by *MET10* and *MET5*, respectively. The lack of a functional *MET10* results in a nonfunctional sulfite reductase, and this shuts down the pathway before the production of homocysteine, *de facto* preventing the cells from producing cysteine and methionine (Fig. 3B) (37).

10.1128/msphere.00393-22.4 TABLE S2 Potentially deleterious heterozygous variants in C. parapsilosis CLIB214. Download Table S2, DOCX file, 0.1 MB. Copyright © 2022 Lombardi et al. 2022 Lombardi et al. https://creativecommons.org/licenses/by/4.0/ This content is distributed under the terms of the Creative Commons Attribution 4.0 International license.

To confirm that *RHA1* does not play a role in sulfur metabolism, we used a different guide RNA to delete the gene (pCP-805700-2) (Fig. 3). All 11 transformants obtained were deleted for *RHA1*, and they could grow in the absence of cysteine and methionine (Fig. S2). By PCR amplifying and sequencing the fragment of *MET10* containing the heterozygous variant, we showed that all the isolates retained both alleles (wild type and stop codon) and that they had a functional copy of Met10, like the parental strain CLIB214 (Fig. S2). Whole-genome sequencing of one representative lineage (805700Δ/Δ) showed that LOH had occurred but not in the region encompassing *MET10*. Instead, the region undergoing LOH extends from the cut site toward the left arm of chromosome 8 (Table 2, Fig. 2B, and Table S1).

10.1128/msphere.00393-22.2 FIG S2 Mutants in which *CPAR2_805700* (*RHA1*) was deleted are cysteine and methionine prototrophic. (A) Amplification of the *CPAR2_805700* (*RHA1*) locus in the 11 transformants (1 to 11, CLIB214 transformed with pCP-805700-2 and delRT-805700-2; Table S5) confirmed the deletion of the gene (primers s805700-FWD [pink arrow] and RhaI-2-dw [blue arrow]; Table S5). The wild-type (WT) allele did not amplify using this primer pair in colony PCR (lane WT), most likely due to its bigger size. (B) Growth with (SC+AA) or without (−Cys/Met) cysteine and methionine. CLIB214 (WT) is included as control. All the transformants are prototrophic for cysteine and methionine. (C) Amplification and Sanger sequencing of the *CPAR2_806380* (*MET10*) locus in the 805700Δ/Δ isolates confirmed that the heterozygous variant (2600C/A) was maintained. The sequencing traces of CLIB214 and one representative transformant are shown. Download FIG S2, TIF file, 2.8 MB. Copyright © 2022 Lombardi et al. 2022 Lombardi et al. https://creativecommons.org/licenses/by/4.0/ This content is distributed under the terms of the Creative Commons Attribution 4.0 International license.

**FIG 4:**
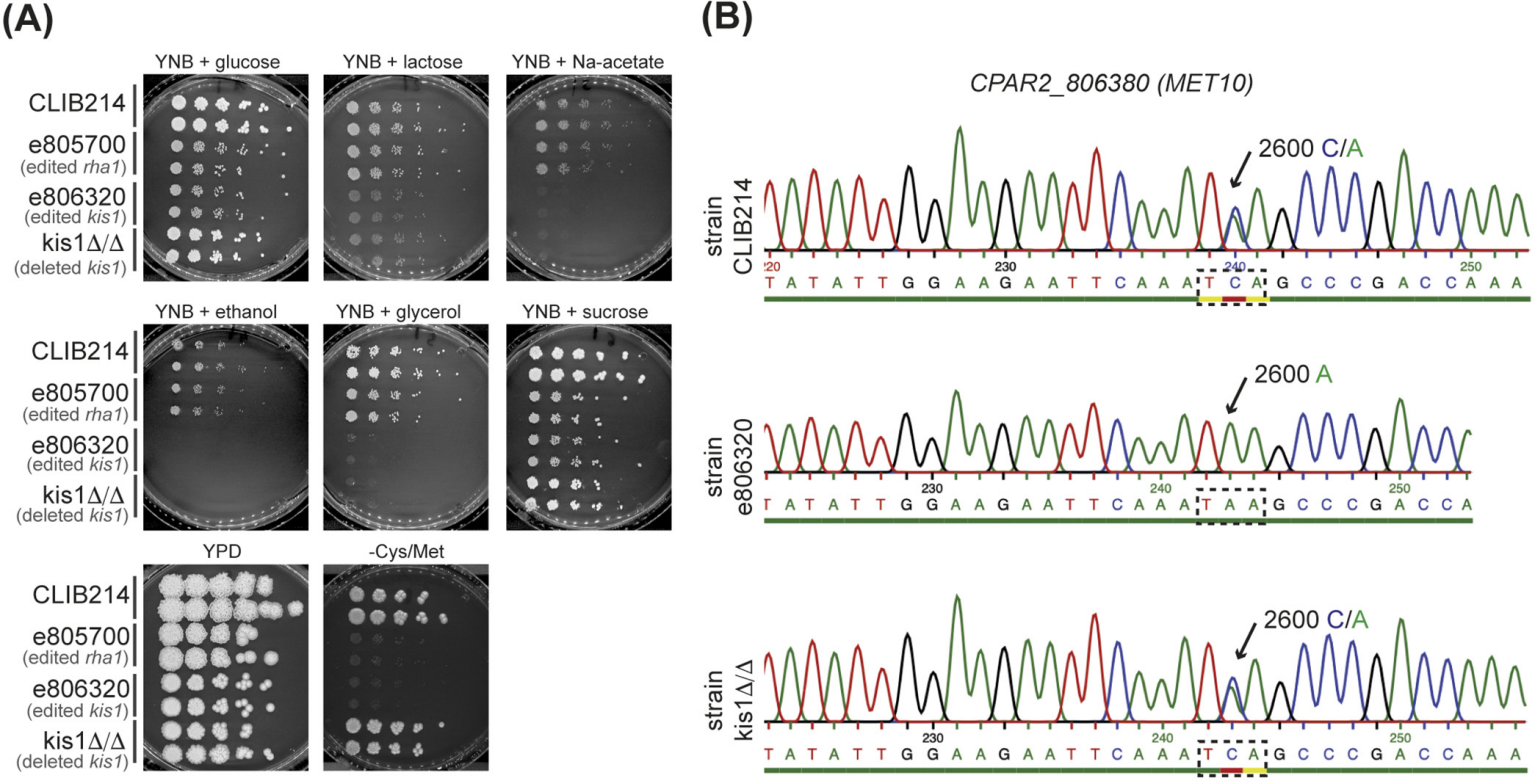
Kis1 is required for utilization of alternative carbon sources but not sulfur metabolism. (A) Growth of parental strain CLIB214 and its derivatives, e805700 (edited *rha1*), e806320 (edited *kis1*), and kisΔ/Δ (deleted *kis1* by allele replacement) (9). The gene targeted by Cas9 is indicated in gray underneath the name of the edited strains. Editing or deleting *KIS1* reduces growth on lactose, sodium acetate, ethanol, and glycerol as sole carbon sources. However, only the edited strain is also auxotrophic for cysteine and methionine, similar to the strain edited in *RHA1* (e805700). (B) Sequencing of the *CPAR2_806380* (*MET10*) locus shows that there is a heterozygous site (C/A in position +2600) in both CLIB214 and kis1Δ/Δ. However, e806320 is homozygous at this position (2600A), showing that both *MET10* alleles in this strain have stop codons.

In an independent experiment, we noticed that disrupting *CPAR2_806320* (*KIS1*) on chromosome 8 also resulted in cysteine and methionine auxotrophy (e806320 in Fig. 4).

In C. albicans, Kis1 is one of the two β subunits of the Snf1p complex, and *KIS1*-deficient mutants fail to grow on many alternative carbon sources (38). As with *RHA1*, no role in sulfur metabolism had been described for *KIS1*. The C. parapsilosis CRISPR-Cas9-edited e806320 strain cannot grow on lactose, sodium acetate, ethanol, or glycerol as sole carbon sources, similar to C. albicans, but it also fails to grow in the absence of cysteine and methionine (Fig. 4). In contrast, a strain in which both *KIS1* alleles were deleted by homologous recombination (806320Δ/Δ) fails to use alternative carbon sources but is a cysteine and methionine prototroph (Fig. 4).

PCR amplification and Sanger sequencing showed that the edited strain e806320 contains stop codons at both *MET10* alleles (Fig. 4). In the deleted strain 806320Δ/Δ, one wild-type allele of *MET10* is retained (Fig. 4). Genome sequencing did not identify a long tract of LOH in e806320 (defined as LOH at a minimum of two adjacent heterozygous sites) (Table 2, Fig. 2B, and Table S1). However, loss of one single heterozygous variant (Chr8, position 1,446,962) 10.7 kb downstream of the cut site, the Ser867/stop codon site in *MET10*, was confirmed. No other LOH was observed in the genome of 806320Δ/Δ (Table 1, Fig. 2A, and Table S1).

### CRISPR-Cas9-associated LOH may result in other unexpected phenotypes.

Our results show that CRISPR-Cas9-induced LOH, and possibly homologous recombination, could cause unplanned phenotypes in edited strains. To determine how widespread the phenomenon could be, we identified all heterozygous sites in C. parapsilosis CLIB214, where homozygosis is predicted to result in deleterious phenotypes. We found 71 heterozygous variants in 67 genes, including 13 stop gains, 6 frameshift mutations, and 52 potentially deleterious nonsynonymous amino acid changes (SIFT score < 0.05; Table S2). Fifty-one genes had orthologs in Saccharomyces cerevisiae (Table S2). The variants are not equally distributed throughout the 8 chromosomes: 26, including 5 stop codons, are located on chromosome 8, reflecting the higher heterozygosity of this chromosome. Overall, the effect of a nonsynonymous amino acid change is often not easy to predict, and therefore, the 52 variants of this type may overestimate the number of potentially deleterious alleles. In contrast, a frameshift mutation or a stop-gain mutation is highly likely to disrupt protein function.

## DISCUSSION

Several studies have shown that replacing alleles of targeted genes by homologous recombination in C. albicans can result in unintended effects, such as aneuploidy and LOH (19–25). Although studies addressing the genome-scale aftermath of CRISPR-Cas9 editing in any *Candida* species are rare, a transient CRISPR-Cas9 system was recently shown to induce both LOH and aneuploidy in C. albicans (31). Here, we compared the genome-wide unplanned effects of classic homologous recombination and CRISPR-Cas9 editing in C. parapsilosis.

We found that some unplanned changes in chromosome number (aneuploidy) in C. parapsilosis manipulated using CRISPR-free homologous recombination (1/9 isolates), CRISPR-Cas9 gene editing (2/12 isolates), and in isolates transformed with a plasmid that expressed Cas9 that was not targeted at a specific gene (2/8 isolates) (Table 1, Table 2, and see Fig. S1 in the supplemental material). The chromosomes affected were not direct targets of gene manipulation (Table 1, Table 2, and Fig. S1), suggesting that, like in C. albicans, transformation is a mutagenic process in C. parapsilosis. Aneuploidies can result in dramatic phenotypic effects (19–22); for example, an extra copy of chromosome 6 in C. parapsilosis drives cross-tolerance to both tunicamycin and aureobasidin A (39).

In C. albicans, LOH can sometimes result in phenotypic changes, such as nitrogen utilization (23) and tolerance to DNA-damaging agents (24). We observed LOH in isolates of C. parapsilosis that were edited using both CRISPR-free homologous recombination and CRISPR-Cas9 editing, but not in isolates that were transformed with only a Cas9-expressing plasmid (Table 1, Table 2, and Table S1). However, LOH events using traditional homologous recombination were rare; LOH was only present in the proximity of the deleted gene in one strain (804640Δ/Δ), possibly connected to homologous recombination at the target site (Table 1; Fig. 2A) (40, 41). All of the isolates obtained by homologous recombination share a 267-kb LOH on the right telomere of chromosome 1. This change is unlikely to be directly associated with deleting *HIS1*, which is on the same chromosome but 15,000 kb upstream of the LOH tract. It must, however, have occurred at some stage during the construction of the his-/leu- derivative C. parapsilosis CPL2H1, which was then used to construct all the deletion strains. However, since only two heterozygous variants, situated 267 kb apart, were lost in this tract of LOH, such unplanned modification is unlikely to have a dramatic effect on the phenotype of the strains (Table S1). In some instances, LOH occurred on chromosomes that were not targeted for editing (Table 1; Table 2).

In contrast with the low frequency of LOH associated with CRISPR-free homologous recombination, LOH was frequently observed in strains that were edited with CRISPR-Cas9 (9/12 isolates), and in most cases (7/9 isolates), it was associated with the targeted chromosome (Table 2, Fig. 2B, and Table S1). Moreover, the LOH tracts observed were large (ranging from 6.5 kb to >1,100 kb), affected several heterozygous sites, and likely reached the telomere in some (4/12 isolates) (Table 2, Fig. 2B, and Table S1). The example of gene *CPAR2_804640* is particularly striking. Deleting both alleles by replacing with *CdHIS1/CmLEU2* resulted in LOH with a maximum length of 8.9 kb (804640Δ/Δ; Table 1; Fig. 2A), whereas editing the same gene using CRISPR-Cas9 (e804640) was associated with LOH extending to the telomere (Table 2, Fig. 2B, and Table S1). We note that the definition of LOH (requiring LOH at a minimum of two adjacent sites) may underestimate the overall level of LOH in CRISPR-edited strains. For example, in strain e806320, no LOH tract was defined, yet it was homozygosis at only one position that resulted in methionine and cysteine auxotrophy (Fig. 2B; Fig. 4).

Most of the LOH that we observed in CRISPR-modified C. parapsilosis isolates started near or at the target locus (Fig. 2): this strongly suggests that LOH may be the result of homology-based repair mechanisms triggered by the Cas9-induced double-strand break (DSB), such as gene conversion (short-tract or long-tract gene conversion events) or break-induced replication (BIR; long-range LOH) (40–42). In two strains (e802210 and 805700Δ/Δ) LOH appears to have occurred on both sides of the Cas9 cut site. However, the length of LOH is more extensive on one side (362 kb compared to 62 kb in strain e802210 and 1,146 kb compared to 8 kb in strain 805700Δ/Δ) (Table 2; Fig. 2B). We also noted that in one strain (805700Δ/Δ), the LOH extended across the centromere of chromosome 8 (Fig. 2B). This may result from more than one independent BIR event or through some other unknown mechanism. The LOH tracts observed do not result from chromosome loss (monosomy) because the sequence coverage is consistent across the genome (except for the aneuploidies described) for all isolates. Overall, we observed LOH on both targeted and nontargeted chromosomes in CRISPR-modified strains of C. parapsilosis.

CRISPR-associated LOH on targeted chromosomes has also been observed in Saccharomyces cerevisiae (30). Whereas Marton et al. (31) suggest that Cas9-associated LOH in C. albicans is rare, we note that transformants were selected based on the acquired double prototrophy (Arg^+^/His^+^), requiring two homology-directed repair (HDR) events with two repair templates, dramatically reducing the likelihood of LOH at the target site. In our experimental design, strains edited at both alleles may be generated by either (i) two HDR events, one between each allele and the repair template (RT) (lower risk of LOH), or (ii) a single HDR event at one allele, followed by a second recombination between the mutated allele and its homolog (higher risk of LOH). LOH events not on the targeted chromosomes were also recently described in C. albicans in strains in which a transient CRISPR-Cas9 system was used to simultaneously integrate two nutritional markers (*ARG4* and *HIS1*) in the two alleles of targeted genes (31).

We found that targeting two different genes on chromosome 8 (*RHA1* and *KIS1*, located approximately 153 and 8 kb upstream of *MET10*, respectively) resulted in cysteine and methionine auxotrophy caused by homozygosity at a single base. Studies from the 1980s using UV treatment to induce mitotic instability in C. albicans noted that auxotrophies were common, and cells with a defect in sulfite reduction (auxotrophic for sulfur-containing amino acids) were particularly enriched (43–45). In 1998, Whelan and Kwon-Chung (46) described a similar phenomenon in C. parapsilosis ATCC 22019 (also known as CLIB214), the parental strain used in this study. We propose that these observations may be explained by UV-induced homozygosis at *MET10*, which has a stop codon in one allele in C. parapsilosis CLIB214.

Despite the risk of frequent and extensive LOH, CRISPR-Cas9 streamlined genetic manipulation of the asexual diploid yeast C. parapsilosis. However, our results show that care must be taken when interpreting ambiguous phenotypic traits of engineered strains. Researchers can—and should—have controls in place to avoid mistakes when associating a mutation with a phenotype. Complementing the mutation introduced is an obvious step to take. Alternatively, LOH could be reduced by integrating two different nutritional markers in the two alleles (as in reference 31). However, this would restrict the use of auxotrophic laboratory strains and lose many of the benefits of markerless constructs. LOH could also be reduced by using two simple repair templates that differ by a few SNPs and screening for the presence of both, one at each allele (as in reference 12). The 71 heterozygous variants that we identified that could potentially result in deleterious phenotypes as a result of LOH in C. parapsilosis CLIB214 constitute a “suspect list” that could be useful in narrowing down a potential culprit for an unexpected phenotype.

### Conclusion.

We showed that Cas9-induced DSB is often associated with LOH in the opportunistic pathogen C. parapsilosis and that this event can be responsible for phenotypic alterations in the engineered strains. We predicted the existence of at least 12 heterozygous variants (frameshifts and stop codons) in the genome of the strain CLIB214 that are likely to be deleterious following homozygosis. Our findings help define the landscape of unplanned genome changes that the CRISPR-Cas9 technology may induce as collateral effects of gene editing in eukaryotic cells with diploid heterozygous genomes and stress the importance of confirming a causal link between the introduced mutation and the observed phenotype.

## MATERIALS AND METHODS

### Strains and media.

All C. parapsilosis strains (see Table S3 in the supplemental material) were grown in YPD medium (1% yeast extract, 2% peptone, and 2% dextrose) or on YPD plates (YPD plus 2% agar) at 30°C. After transformation with pCP-tRNA, transformants were selected on YPD agar supplemented with 200 μg/mL nourseothricin (Werner Bioagents, Jena, Germany). Deletion strains that were constructed in the CPL2H1 background were selected on the appropriate dropout agar plates as described in reference 9. Plasmids (Table S5) were propagated in Escherichia coli DH5α cells (NEB, United Kingdom) by growing cells in LB media without NaCl (formedium) supplemented with 100 μg/mL ampicillin (Sigma).

10.1128/msphere.00393-22.5 TABLE S3 Strains used in this study. Download Table S3, DOCX file, 0.01 MB. Copyright © 2022 Lombardi et al. 2022 Lombardi et al. https://creativecommons.org/licenses/by/4.0/ This content is distributed under the terms of the Creative Commons Attribution 4.0 International license.

### Phenotypic testing and spot assay.

Auxotrophies were identified by growing mutant strains on synthetic complete (SC) dropout medium (0.19% yeast nitrogen base without amino acids and ammonium sulfate, 0.5% ammonium sulfate, 2% glucose, 0.075% cysteine-methionine dropout mix, and 2% agar). The utilization of different carbon sources was tested on yeast nitrogen base without ammonium sulfate (YNB; 0.19%) supplemented with 0.5% ammonium sulfate and either 2% glucose or 2% alternative carbon sources. For the spot assays, overnight cultures were grown in YPD at 30°C with shaking, washed, and diluted to a final optical density at 600 nm (OD_600_) of 0.0625 in 1 mL of phosphate-buffered saline (PBS). The cultures were serially diluted (1:5) in a 96-well microtiter plate and then spotted on phenotyping plates with a 48-pin bolt replicator. The plates were grown at 30°C for 2 days and then photographed.

### Construction of deletion strains in Candida parapsilosis with a CRISPR-free homologous recombination method.

Six isolates sequenced in this study were generated using a CRISPR-free method previously published (9). Deletion strains were generated by fusion PCR in C. parapsilosis CPL2H1, a *leu2 his1* double auxotrophic strain derived from C. parapsilosis CLIB214 (9). The oligonucleotides used to synthesize the deletion cassettes and to confirm the integrations are listed in Table S4. Transformation was performed as in reference 9. Three additional isolates generated in reference 9 were included in the sequencing.

10.1128/msphere.00393-22.6 TABLE S4 Oligonucleotides used for CRISPR-free gene deletion. Download Table S4, DOCX file, 0.02 MB. Copyright © 2022 Lombardi et al. 2022 Lombardi et al. https://creativecommons.org/licenses/by/4.0/ This content is distributed under the terms of the Creative Commons Attribution 4.0 International license.

### Gene editing using pCP-tRNA in Candida parapsilosis.

The oligonucleotides used to generate and screen the edited mutants are listed in Table S5. The edited strains were constructed by CRISPR-Cas9 editing in C. parapsilosis CLIB214 using the pCP-tRNA plasmid (12), available at Addgene (plasmid number 133812). Suitable guides to induce Cas9 cleavage were computationally designed with EuPaGDT (47) and cloned into the SapI-digested pCP-tRNA plasmid as described in reference 12. The presence of the guide in the receiving plasmid was confirmed by PCR (M13FWD universal primer with relevant gRNA_BOT oligonucleotide [Table S5]). Repair templates (RTs) for editing (eRTs) were designed to repair the Cas9-induced double-strand break (DSB) by homology-directed repair (HDR), containing 30-bp homology arms to either side of the cut, 11-bp introducing stop codons in all three reading frames, and a unique 20-bp barcode (tag). For the edited mutant e301940, the RT contains 40-bp homology arms on either side of the cut, two in-frame stop codons (6 bp), and a unique barcode (Table S5). Each eRT was generated by primer extension as described in reference 12 (Table S5). To delete *CPAR2_805700*, two different 1,020-bp-long deletion RTs (delRTs) generated by fusion PCR were used, aimed at inducing either (i) the replacement of the entire ORF with the barcode, or (ii) the replacement of a 1248-bp-long central region of the ORF (+1998/+3245, after amino acid 259) with the barcode (Table S5). Three to five micrograms of purified delRT were used to transform C. parapsilosis in combination with the relevant pCP-tRNA plasmid. All oligonucleotides were ordered from Eurofins Genomics. Two independent disrupted strains (A and B) were constructed for each gene target. The names of the edited strains (e.g., e301940) reflect the target gene (e.g., *CPAR2_301940*). C. parapsilosis CLIB214 was also transformed with pCP-tRNA plasmid with no guide RNA, and the genomes of eight transformants growing on selective plates were sequenced as control (transformants 1 to 8).

### Transformation of Candida parapsilosis.

Yeast cells were transformed with 5 μg of the relevant plasmid and either 25 μL of unpurified repair template for editing the gene (eRT) or 5 μg of purified repair template for deleting the gene (delRT), using the lithium acetate method described in reference 48, with minor modifications (starting OD_600_ of YPD culture of 0.1 instead of 0.05). Transformants were selected onto YPD agar plates containing 200 μg/mL nourseothricin (Jena Bioscience GmbH, Germany) and screened by colony PCR to confirm the presence of the mutation (Table S4 and Table S5). Representative mutants were sequenced by Sanger sequencing (MWG/Eurofins). For each mutant strain, two independent lineages (A and B) were patched onto YPD agar without selection twice to induce the loss of the pCP-tRNA plasmid.

### DNA extraction for whole-genome sequencing.

The genome DNA of one isolate from each targeted gene disruption or deletion was sequenced. Cells were grown overnight in YPD with shaking, harvested by centrifugation (3,000 rpm, 5 min), and resuspended in 200 μL of extraction buffer (2% [mass/vol] Triton X-100, 100 mM NaCl, 10 mM Tris, pH 7.4, 1 mM EDTA, and 1% [mass/vol] SDS) and 200 μL of phenol-chloroform-isoamyl alcohol (25:24:1) (PCIA). Cells were lysed by adding 0.3-g acid-washed glass beads (0.45 to 0.52 mm; Sigma) and agitating the mixture with a 600 MiniG bead beater (Spex Sample Prep) for 30 s (6 times).

The mixture was centrifuged (14,000 rpm, 10 min), and the aqueous phase was transferred into a new tube. The aqueous phase was then extracted by adding 200 μL Tris-EDTA (TE) buffer (pH 8.0) and 200 μL PCIA to the tube and agitating in the bead beater (30 s), followed by centrifugation and a second treatment with TE-PCIA and a third with 200 μL PCIA only.

Nucleic acids were precipitated by adding 1 mL 100% isopropanol with 80 μL 7.5 M ammonium acetate to the aqueous layer and pelleted by centrifugation. The pellet was washed with 1 mL of 70% ethanol, air-dried, resuspended in 400 μL TE buffer and 1 μL RNase A (100 mg/mL), and incubated at 37°C for 1 h. DNA was reprecipitated and washed as above and resuspended in 100 μL of deionized water. The DNA was then cleaned with the genomic DNA Clean and Concentrator-10 kit (Zymo Research) according to the manufacturer’s instructions.

### Genome sequencing.

For strains e101740, e205070, e301780, e301940, e802210, e804640, e804830, e804940, e805700, and del805700, whole-genome sequencing was performed by Beijing Genomics Institute (BGI) on a DNBseq platform generating 150-bp paired-end reads. Strains 302230Δ/Δ, 302310Δ/Δ, 802440Δ/Δ, 804640Δ/Δ, 203540Δ/Δ, 802880Δ/Δ, 805000Δ/Δ, 803920Δ/Δ, 805700Δ/Δ, e806320, and 806320Δ/Δ and the 8 isolates transformed with the pCP-tRNA plasmid were sequenced in-house. To prepare the libraries, 500 ng of genomic DNA was quantified using a fluorometric method (Qubit broad range [BR]), tagmented, and amplified using the Illumina DNA prep kit (catalog no. 20018704) following the manufacturer’s instructions. Quality control of amplified libraries was performed using Bioanalyzer DNA high-sensitivity (HS) chip (Agilent Technologies). Finally, an Illumina NextSeq 500 platform was used to perform paired-end sequencing of the samples with ID output kit v.2.5 (300 cycles; Illumina).

Read depth was calculated by dividing the raw amount of bases derived in the sequencing run by the approximate size (13 Mb) of the C. parapsilosis reference genome (CDC317). Sequencing depth ranged from ~23× to ~71× across the sample set.

All reads were trimmed using Skewer (v.0.2.2) (49) to minimum lengths of 30 and average qualities of 35. Trimmed reads were aligned to the C. parapsilosis CDC317 reference using BWA-MEM (v.0.7.17-r1188) (50). Sorting and duplicate marking were performed using samtools sort (v.1.10) and Picard Tools (v.2.21.6), respectively, on output BAM files (51). The Genome Analysis Tool kit (GATK v.4.2.0.0) was used to call variants per sample in GVCF format, combining records and joint genotypes (52). GATK VariantFiltration was used to filter out variants below a read depth of 15 and minimum genotype quality of 40. Clusters of 5 SNPs in 100-bp windows were removed. Variants flanked by long mono- or dinucleotide repeats were removed using a custom script (https://github.com/CMOTsean/milt_variant_filtration). Heterozygous alleles with a depth ratio of below 0.25 or above 0.75 were also removed (53). Sites for which genotypes could not be called in three or more strains (“nocall”) were removed. To identify LOH ranges, sites which were heterozygous in C. parapsilosis CLIB214 were extracted (Table S1). LOH was defined as a minimum of two adjacent sites that are heterozygous in CLIB214 and homozygous in at least one of the edited strains. For chromosome 8, these sites were plotted as vertical lines along the chromosome using the Matplotlib Python package (54).

Sample coverage and chromosomal copy number were analyzed using BEDTools and Delly (55, 56). Annotation of gene variants and prediction of protein-coding effects were performed using SIFT. A SIFT4G database for C. parapsilosis CDC317 was generated for Candida parapsilosis as described in references 53 and 57.

### Data availability.

The data generated in this study have been submitted to the NCBI BioProject database (https://www.ncbi.nlm.nih.gov/bioproject/) under accession number PRJNA866533.
