# Supplementary figures and images for: CRISPR-Cas9 Editing Induces Loss of Heterozygosity in the Pathogenic Yeast Candida parapsilosis
Source: mSphere. 2022 Nov 23;7(6):e00393-22. doi: 10.1128/msphere.00393-22 (PMC9769790; doi:10.1128/msphere.00393-22)

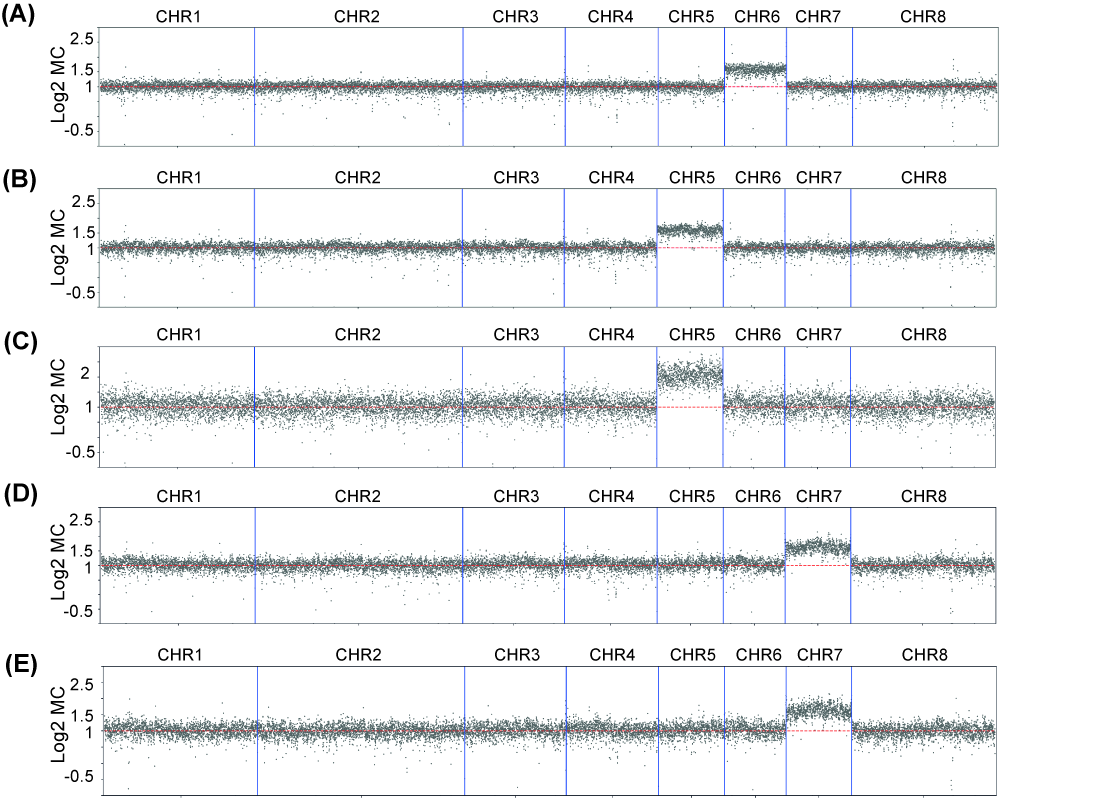

Supplement: FIG S1 [file msphere.00393-22-s0001.tif]

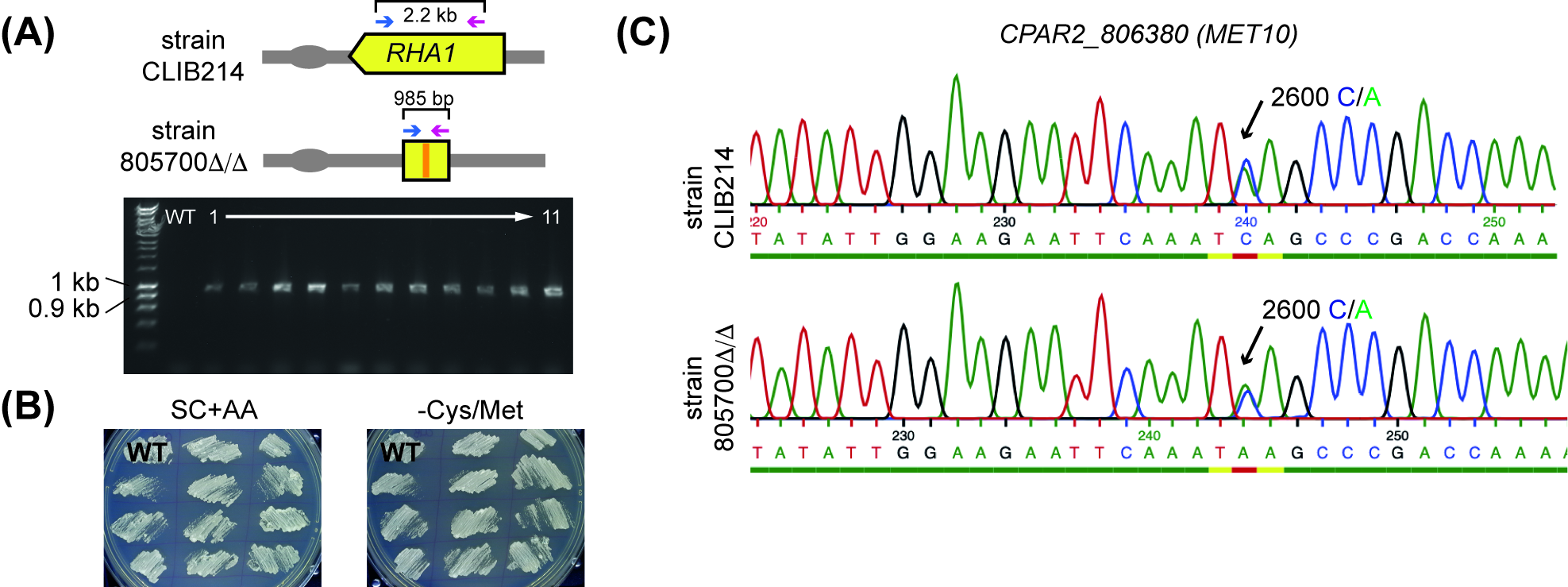

Supplement: FIG S2 [file msphere.00393-22-s0002.tif]
