# Supplementary material for: CRISPR-Cas9 Editing Induces Loss of Heterozygosity in the Pathogenic Yeast Candida parapsilosis
Source: mSphere. 2022 Nov 23;7(6):e00393-22. doi: 10.1128/msphere.00393-22 (PMC9769790; doi:10.1128/msphere.00393-22)
Supplement: TABLE S5 [file msphere.00393-22-s0007.docx]

**Table S5. Plasmids and oligonucleotides used for CRISPR-Cas9 modifications.** For each strain, the oligonucleotides used for generating the relevant pCP-tRNA plasmid and Repair Templates (RTs) are indicated in the Table, as well as the oligonucleotides used for screening the strains and sequencing the mutated locus. In the sequence of the RT, the barcode and the 11-nt sequence resulting in the introduction of one in-frame stop codon are highlighted in yellow and red, respectively. The plasmids used for introducing the Cas9-mediated cut are indicated in bold. Note that the edited mutant e301940 was generated at an early stage of the construction of the library, and the RT contains two in-frame stop codon (6 bp) and the unique barcode. The primers for sequencing the *CPAR2_806380* (*MET10*) allele in different strains are reported at the end of the table.

| **Strain** | **Name oligos** | **sequence (5'-3')** | **Function** |
| --- | --- | --- | --- |
| e805700_A/B | g805700_TOP | CCACAATTCGTCCACATTGTTGG | cloning of the guide 1 targeting CPAR2_805700 into SapI-digested pCP-tRNA to generate **pCP-805700 (pCP-RHAI)** |
|  | g805700_BOT | AACCCAACAATGTGGACGAATTG |  |
|  | RT805700_TOP | TGGATTCGTCTCCATATTCTGACTCCCCCATAGATAGATAGCCCGCGTGTCGATTA | synthesis of eRT-805700 by primer extension; this RT introduces an in-frame premature stop codon and a barcode in the cut site |
|  | RT805700_BOT | AGTAACTAGAAAACAATTCGTCCACATTGTAAACCTAATCGACACGCGGGCTATC |  |
|  | p805700_FWD | CCCGCGTGTCGATTAGGTTT | screening of the transformants (the FWD primer anneals on the unique barcode) |
|  | p805700_REV | CGTAATCAGAACTATCGTGG |  |
|  | S805700_FWD | GGTCGTAACCATAATCAACC | Sequencing of the edited locus |
|  | p805700_REV | As above |  |
| “del”805700_A/B | g805700_TOP | As above | cloning of the guide 1 targeting CPAR2_805700 into SapI-digested pCP-tRNA to generate **pCP-805700 (pCP-RHAI)** |
|  | g805700_BOT | As above |  |
|  | 805700-up | ATTATGGGGTTCAATGGAGG | synthesis of delRT-805700 by fusion PCR; this RT is designed to replace the gene with the barcode |
|  | 805700-dw | ATGCGTTAACACCGAAAAAC |  |
|  | 805700-TAG-fwd | CCCGCGTGTCGATTAGGTTTACAGTAGAGCAAATCTTACA |  |
|  | 805700-TAG-rev | AAACCTAATCGACACGCGGGGTTACACTCTATAATGGTG |  |
| 805700Δ/Δ_A/B | g805700-2-TOP | CCAAATCACGCTAGGAGAGGCTT | cloning of the guide 2 targeting CPAR2_805700 into SapI-digested pCP-tRNA to generate **pCP-805700-2 (pCP-RHAI-2)** |
|  | g805700-2-BOT | AACAAGCCTCTCCTAGCGTGATT |  |
|  | s805700-FWD | GGTCGTAACCATAATCAACC | synthesis of delRT-805700-2 by fusion PCR; HDR with the RT results in the deletion of 1248 bp surrounding the cut site in the central portion of the gene, and the introduction of the barcode |
|  | RhaI-2-TAG-R | AAACCTAATCGACACGCGGGCCAAGTGGAGCGTTATTGAA |  |
|  | RhaI-2-TAG-F | CCCGCGTGTCGATTAGGTTTGTCTCCACTCAAAAGAACAT |  |
|  | RhaI-2-dw | TGGTTGTGATTCTCACGCTC |  |
| e806320_A/B | g806320_TOP | CCAGAACCTGATCAAGAGGGAAG | cloning of the guide targeting CPAR2_806320 into SapI-digested pCP-tRNA to generate **pCP-806320** |
|  | g806320_BOT | AACCTTCCCTCTTGATCAGGTTC |  |
|  | RT806320_TOP | TATCGACAGAAAAGAACCTGATCAAGAGGGTAGATAGATAGAATAGCCTTCAACGT | synthesis of eRT-806320 by primer extension; this RT introduces an in-frame premature stop codon and a barcode in the cut site |
|  | RT806320_BOT | TCACCACCAACACCAGCATCACCTCCACTTGCTAGACGTTGAAGGCTATTCTATC |  |
|  | p806320_FWD | AATAGCCTTCAACGTCTAGC | screening of the transformants (the FWD primer anneals on the unique barcode) |
|  | p806320_REV | CCCATCTGGCTGTTTAACTA |  |
|  | s806320_FWD | GTGTCTATGGTTGATGTGAG | Sequencing of the edited locus |
|  | p806320_REV | As above |  |
| e101740_A/B | g101740_TOP | CCAATTGAGGAGTACGTTAAAAG | cloning of the guide targeting CPAR2_101740 into SapI-digested pCP-tRNA to generate **pCP-101740** |
|  | g101740_BOT | AACCTTTTAACGTACTCCTCAAT |  |
|  | RT101740_TOP | TCAAACCCAAAGTATTGAGGAGTACGTTAATAGATAGATAGACCGGACGGAGCTAT | synthesis of eRT-101740 by primer extension; this RT introduces an in-frame premature stop codon and a barcode in the cut site |
|  | RT101740_BOT | ATATAAAATCCTTCCACCTTCAATCCGCTTCAAATATAGCTCCGTCCGGTCTATC |  |
|  | p101740_FWD | ACCGGACGGAGCTATATTTG | screening of the transformants (the FWD primer anneals on the unique barcode) |
|  | p101740_REV | TTCCCGAGATAGTAGTTTGC |  |
|  | s101740_FWD | GGAGACAGAGAGCAAAATAG | Sequencing of the edited locus |
|  | p101740_REV | As above |  |
| e205070_A/B | g205070_TOP | CCAGAGCGAGACACATTTGGTGA | cloning of the guide targeting CPAR2_205070 into SapI-digested pCP-tRNA to generate **pCP-205070** |
|  | g205070_BOT | AACTCACCAAATGTGTCTCGCTC |  |
|  | RT205070_TOP | GTTTCATCCATCCCATCAAGTAGACCATCATAGATAGATAGCTTCATAGTGTGACA | synthesis of eRT-205070 by primer extension; this RT introduces an in-frame premature stop codon and a barcode in the cut site |
|  | RT205070_BOT | CTTTCTTGATGACGAGCGAGACACATTTGGATCCCTGTCACACTATGAAGCTATC |  |
|  | p205070_FWD | CTTCATAGTGTGACAGGGAT | screening of the transformants (the FWD primer anneals on the unique barcode) |
|  | p205070_REV | CTGGTGTGGTTCTTTACTTG |  |
|  | s205070_FWD | CTCACAAGTCATCACCTTTG | Sequencing of the edited locus |
|  | p205070_REV | As above |  |
| e301780_A/B | g301780_TOP | CCAGGATGTTCTTGACTCGTTCG | cloning of the guide targeting CPAR2_301780 into SapI-digested pCP-tRNA to generate **pCP-301780** |
|  | g301780_BOT | AACCGAACGAGTCAAGAACATCC |  |
|  | RT301780_TOP | TGGTTCAAAAAGAGGATGTTCTTGACTCGTTAGATAGATAGATGTGTATGCCACTA | synthesis of eRT-301780 by primer extension; this RT introduces an in-frame premature stop codon and a barcode in the cut site |
|  | RT301780_BOT | AGGCGTCGTCTATAGTATCGGTTGCCTCGACGCGGTAGTGGCATACACATCTATC |  |
|  | p301780_FWD | ATGTGTATGCCACTACCGCG | screening of the transformants (the FWD primer anneals on the unique barcode) |
|  | p301780_REV | CCTGACAAGAAGTACCTTAC |  |
|  | s301780_FWD | CAAGAAAGAGAGAGAGAGAG | Sequencing of the edited locus |
|  | p301780_REV | As above |  |
| e301940_A/B | g301940_TOP | CCAAATAAACAGAACCGATTTGG | cloning of the guide targeting CPAR2_301940 into SapI-digested pCP-tRNA to generate **pCP-301940** |
|  | g301940_BOT | AACCCAAATCGGTTCTGTTTATT |  |
|  | RT301940_TOP | ACAACAACACACAGGTAACAAATAATAAACAGAACCGATTTTAATAGATTACTCTACGCAGCGG | synthesis of eRT-301940 by primer extension; this RT introduces an in-frame premature stop codon and a barcode in the cut site |
|  | RT301940_BOT | ATTCTTTGTATCTTGATGATGATGATAATGGTTACCACCACCTCCGCTGCGTAGAGTAATCTA |  |
|  | p301940_FWD | ATTACTCTACGCAGCGGAGG | screening of the transformants (the FWD primer anneals on the unique barcode) |
|  | p301940_REV | GAAATCCTGGTGATGGCTGT |  |
|  | s301940_FWD | TGTTGTTGTTGTTGGGTGTC | Sequencing of the edited locus |
|  | p301940_REV | As above |  |
| e802210_A/B | g802210_TOP | CCATTGGCATAATCATGCAAAGA | cloning of the guide targeting CPAR2_802210 into SapI-digested pCP-tRNA to generate **pCP-802210** |
|  | g802210_BOT | AACTCTTTGCATGATTATGCCAA |  |
|  | RT802210_TOP | ATTAAAACCATGAAGAAAAAGCTTCCATCTTAGATAGATAGCATAGAGTTCGAGAC | synthesis of eRT-802210 by primer extension; this RT introduces an in-frame premature stop codon and a barcode in the cut site |
|  | RT802210_BOT | GATTTCTTTCACTTTGGCATAATCATGCAACGGCTGTCTCGAACTCTATGCTATC |  |
|  | p802210_FWD | CATAGAGTTCGAGACAGCCG | screening of the transformants (the FWD primer anneals on the unique barcode) |
|  | p802210_REV | GTAGTCTCCCAACTTGATGA |  |
|  | s802210_FWD | ACCTTCTGTCACTATCTTCC | Sequencing of the edited locus |
|  | p802210_REV | As above |  |
| e804640_A/B | g804640_TOP | CCAAAAATGCATTTAACCAATCG | cloning of the guide targeting CPAR2_804640 into SapI-digested pCP-tRNA to generate **pCP-804640** |
|  | g804640_BOT | AACCGATTGGTTAAATGCATTTT |  |
|  | RT804640_TOP | CTCAGTCAAGTCAAGCAATGAGTACCTCGATAGATAGATAGAGGTAATATACACTC | synthesis of eRT-804640 by primer extension; this RT introduces an in-frame premature stop codon and a barcode in the cut site |
|  | RT804640_BOT | GGACATTTACTCGAAAATGCATTTAACCAAGCGGTGAGTGTATATTACCTCTATC |  |
|  | p804640_FWD | AGGTAATATACACTCACCGC | screening of the transformants (the FWD primer anneals on the unique barcode) |
|  | p804640_REV | CATATTGACTTGGGGTGAAG |  |
|  | s804640_FWD | GAATATGAACGATCGCTCAC | Sequencing of the edited locus |
|  | p804640_REV | As above |  |
| e804830_A/B | g804830_TOP | CCAATATAGGTCCCAGTGATTGT | cloning of the guide targeting CPAR2_804830 into SapI-digested pCP-tRNA to generate **pCP-804830** |
|  | g804830_BOT | AACACAATCACTGGGACCTATAT |  |
|  | RT804830_TOP | AGCTACAGATCCAGACAGCGAAAACCAACATAGATAGATAGCCATAGGTTTAACGC | synthesis of eRT-804830 by primer extension; this RT introduces an in-frame premature stop codon and a barcode in the cut site |
|  | RT804830_BOT | TGACAATTATCACATATAGGTCCCAGTGATCGCAAGCGTTAAACCTATGGCTATC |  |
|  | p804830_FWD | CCATAGGTTTAACGCTTGCG | screening of the transformants (the FWD primer anneals on the unique barcode) |
|  | p804830_REV | GTCGTAGTCTCTTGATGATG |  |
|  | s804830_FWD | GTCTGGAATACTACCCTTTG | Sequencing of the edited locus |
|  | p804830_REV | As above |  |
| e804940_A/B | g804940_TOP | CCAGTCATCGGAGTCGTCACCAG | cloning of the guide targeting CPAR2_804940 into SapI-digested pCP-tRNA to generate **pCP-804940** |
|  | g804940_BOT | AACCTGGTGACGACTCCGATGAC |  |
|  | RT804940_TOP | CGATTATTGAAAATGAGAACAACTCCACTGTAGATAGATAGCCATCGTTCACAATC | synthesis of eRT-804940 by primer extension; this RT introduces an in-frame premature stop codon and a barcode in the cut site |
|  | RT804940_BOT | CATCGGCATCAAAGTCATCGGAGTCGTCACTCCTAGATTGTGAACGATGGCTATC |  |
|  | p804940_FWD | CCATCGTTCACAATCTAGGA | screening of the transformants (the FWD primer anneals on the unique barcode) |
