# Supplementary material for: CRISPR-Cas9 Editing Induces Loss of Heterozygosity in the Pathogenic Yeast Candida parapsilosis
Source: mSphere. 2022 Nov 23;7(6):e00393-22. doi: 10.1128/msphere.00393-22 (PMC9769790; doi:10.1128/msphere.00393-22)
Supplement: TABLE S2 [file msphere.00393-22-s0004.docx]

**Table S2. Potentially deleterious heterozygous variants in *C. parapsilosis* CLIB214.** FD: frameshift deletion; FI: frameshift insertion; NS: non-synonymous.

**
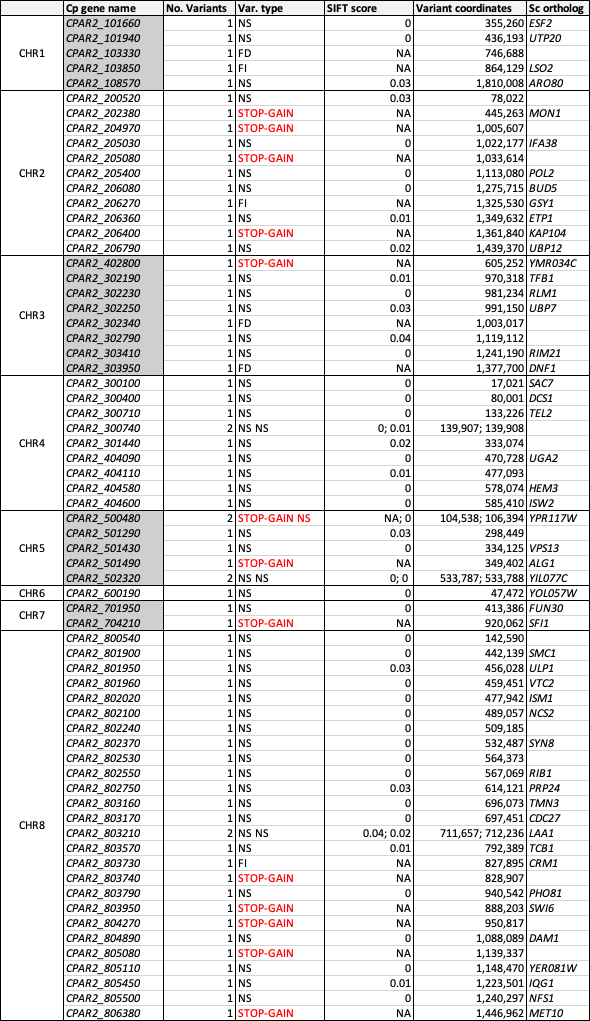
**
