# Supplementary material for: CRISPR-Cas9 Editing Induces Loss of Heterozygosity in the Pathogenic Yeast Candida parapsilosis
Source: mSphere. 2022 Nov 23;7(6):e00393-22. doi: 10.1128/msphere.00393-22 (PMC9769790; doi:10.1128/msphere.00393-22)
Supplement: TABLE S3 [file msphere.00393-22-s0005.docx]

**Table S3. Strains used in this study.** The letters A and B represent the two different lineages of a strain.

| **Strain** | **Genotype** | **Method** | **Modification of**  **target gene** | **Reference** |
| --- | --- | --- | --- | --- |
| CLIB214 |  |  |  | Type Strain |
| CPH1L2 | *leu2::FRT/leu2::FRT, his1::FRT/his1::FRT* | SAT flipper | deletion | (10) |
| e101740_A/B | *cpar2_101740::StopTAG/ cpar2_101740::StopTAG* | CRISPR-Cas9 | stop codon | This study |
| e205070_A/B | *cpar2_205070::StopTAG/ cpar2_205070::StopTAG* | CRISPR-Cas9 | stop codon | This study |
| e301780_A/B | *cpar2_301780::StopTAG/ cpar2_301780::StopTAG* | CRISPR-Cas9 | stop codon | This study |
| e301940_A/B | *cpar2_301940::StopTAG/ cpar2_301940::StopTAG* | CRISPR-Cas9 | stop codon | This study |
| e802210_A/B | *cpar2_802210::StopTAG/ cpar2_802210::StopTAG* | CRISPR-Cas9 | stop codon | This study |
| e804640_A/B | *cpar2_804640::StopTAG/ cpar2_804640::StopTAG* | CRISPR-Cas9 | stop codon | This study |
| e804830_A/B | *cpar2_804830::StopTAG/ cpar2_804830::StopTAG* | CRISPR-Cas9 | stop codon | This study |
| e804940_A/B | *cpar2_805700::StopTAG/ cpar2_805700::StopTAG* | CRISPR-Cas9 | stop codon | This study |
| e805700_A/B | *cpar2_804940::StopTAG/ cpar2_804940::StopTAG* | CRISPR-Cas9 | stop codon | This study |
| “del”805700_A/B | *cpar2_805700/cpar2_805700* | CRISPR-Cas9 | none | This study |
| 805700 Δ/Δ _A/B | *cpar2_805700::TAG/ cpar2_805700::TAG* | CRISPR-Cas9 | deletion | This study |
| e806320_A/B | *cpar2_806320::StopTAG/ cpar2_806320::StopTAG* | CRISPR-Cas9 | stop codon | This study |
| kis1Δ/Δ _A/B | *leu2::FRT/leu2::FRT, his1::FRT/his1::FRT, kis1::LEU2/kis1::HIS1* | HIS1/LEU2 | deletion | (10) |
| cla4Δ/Δ_A/B | *leu2::FRT/leu2::FRT, his1::FRT/his1::FRT, cla4::LEU2/cla4::HIS1* | HIS1/LEU2 | deletion | (10) |
| wor1Δ/Δ_A/B | *leu2::FRT/leu2::FRT, his1::FRT/his1::FRT, wor1::LEU2/wor1::HIS1* | HIS1/LEU2 | deletion | (10) |
| 803920 Δ/Δ_A/B | *leu2::FRT/leu2::FRT, his1::FRT/his1::FRT, cpar2_803920::LEU2/cpar2_803920::HIS1* | HIS1/LEU2 | deletion | This study |
| 101530 Δ/Δ_A/B | *leu2::FRT/leu2::FRT, his1::FRT/his1::FRT, cpar2_101530::LEU2/cpar2_101530::HIS1* | HIS1/LEU2 | deletion | This study |
| 802440 Δ/Δ_A/B | *leu2::FRT/leu2::FRT, his1::FRT/his1::FRT, cpar2_802440::LEU2/cpar2_802440::HIS1* | HIS1/LEU2 | deletion | This study |
| 203540 Δ/Δ_A/B | *leu2::FRT/leu2::FRT, his1::FRT/his1::FRT, cpar2_203540::LEU2/cpar2_203540::HIS1* | HIS1/LEU2 | deletion | This study |
| 204950 Δ/Δ_A/B | *leu2::FRT/leu2::FRT, his1::FRT/his1::FRT, cpar2_204950::LEU2/cpar2_204950::HIS1* | HIS1/LEU2 | deletion | This study |
| 302310 Δ/Δ_A/B | *leu2::FRT/leu2::FRT, his1::FRT/his1::FRT, cpar2_302310::LEU2/cpar2_302310::HIS1* | HIS1/LEU2 | deletion | This study |
| 802880 Δ/Δ_A/B | *leu2::FRT/leu2::FRT, his1::FRT/his1::FRT, cpar2_802880::LEU2/cpar2_802880::HIS1* | HIS1/LEU2 | deletion | This study |
| 302230 Δ/Δ_A/B | *leu2::FRT/leu2::FRT, his1::FRT/his1::FRT, cpar2_302230::LEU2/cpar2_302230::HIS1* | HIS1/LEU2 | deletion | This study |
