# Supplementary material for: CRISPR-Cas9 Editing Induces Loss of Heterozygosity in the Pathogenic Yeast Candida parapsilosis
Source: mSphere. 2022 Nov 23;7(6):e00393-22. doi: 10.1128/msphere.00393-22 (PMC9769790; doi:10.1128/msphere.00393-22)
Supplement: TABLE S4 [file msphere.00393-22-s0006.docx]

**Table S4. Oligonucleotides used for CRISPR-free gene deletion.** The Table includes the oligonucleotides used for constructing the mutants (Primers 1 – 6) and checking the 5’ and 3’ correct integration of the cassettes (5'check + His1-check-Rv; HIS2-check-Fv + 3'check); (5'check + Leu1-check-Rv; Leu2-check-Fv + 3'check). Primers are named as in (10).

| **Universal Primers** | | | | |
| --- | --- | --- | --- | --- |
| HIS1-check Rv | | AAAATCAATGGGCATTCTCG | | |
| HIS2-check Fw | | TGGGAAGCAGACATTCAACA | | |
| LEU1-check Rv | | GAAGTTGGTGACGCGATTGT | | |
| LEU2-check Fw | | TTCCCCTTCAATGTATGCAA | | |
| **Strain** | **Primer 1** | **Primer 2** | **Primer 3** | **Primer 5** |
| **803920 Δ/Δ_A/B** | GGTGTACAATGGTAACATTG | cacggcgcgcctagcagcggTTTGCGAATGGATATAAATC | ccgctgctaggcgcgccgtgACCAGTGTGATGGATATCTGC | gcagggatgcggccgctgacCCAAATAAAGCATGGTCGCCagctcggatccactagtaacg |
| **101530 Δ/Δ_A/B** | AGGATTGTGTGTTGGGGTGG | cacggcgcgcctagcagcggTGATTGTCAAATTTGTTTGT | ccgctgctaggcgcgccgtgACCAGTGTGATGGATATCTGC | gcagggatgcggccgctgacACCCTACATGGGATGAAGAGagctcggatccactagtaacg |
| **802440 Δ/Δ_A/B** | TTTTAAAGTGCTCTCTTGTC | cacggcgcgcctagcagcggTGTGAATTATAATGTGGAAT | ccgctgctaggcgcgccgtgACCAGTGTGATGGATATCTGC | gcagggatgcggccgctgacCATAGTGGACAAGTACGTGAagctcggatccactagtaacg |
| **203540 Δ/Δ_A/B** | TTTGTCTCCTATTTTTTTCG | cacggcgcgcctagcagcggGATTGCAAAAGGATTCGTGG | ccgctgctaggcgcgccgtgACCAGTGTGATGGATATCTGC | gcagggatgcggccgctgacCTATCACGGGTCAGGAGTCTagctcggatccactagtaacg |
| **204950 Δ/Δ_A/B** | AAGTGGTAAATGCTCGACCT | cacggcgcgcctagcagcggAGAGAAGGAGTTATGTGGGG | ccgctgctaggcgcgccgtgACCAGTGTGATGGATATCTGC | gcagggatgcggccgctgacCTTACTGTAGGAGAGAGACTagctcggatccactagtaacg |
| **302310 Δ/Δ_A/B** | AGATGGAAGCTTGCTTACAG | cacggcgcgcctagcagcggCTCCCTATAGTAATCTATAT | ccgctgctaggcgcgccgtgACCAGTGTGATGGATATCTGC | gcagggatgcggccgctgacATTCAGCTCAGTCTGCACATagctcggatccactagtaacg |
| **802880 Δ/Δ_A/B** | TTCGAGACAATGCAGGGGTA | cacggcgcgcctagcagcggTCTCCGTAAAGAGTAGTCAT | ccgctgctaggcgcgccgtgACCAGTGTGATGGATATCTGC | gcagggatgcggccgctgacCATCGCGTGTCAGTGCTTGTagctcggatccactagtaacg |
| **302230 Δ/Δ_A/B** | ATCTGCCACCAAAACCCTAT | cacggcgcgcctagcagcggAGTAACTTCTTTTTTCTCTT | ccgctgctaggcgcgccgtgACCAGTGTGATGGATATCTGC | gcagggatgcggccgctgacATTATTGTCCCAGGCGCTCGagctcggatccactagtaacg |

| **Strain** | **Primer 4** | **Primer 6** | **5' Check-Fw** | **3' Check-Rv** | **ORF check-Rv** |
| --- | --- | --- | --- | --- | --- |
| **803920 Δ/Δ_A/B** | gtcagcggccgcatccctgcTAGTTTCAATCATCAATCAA | ACCCAATTTTTATCAAAGAT | TTGACAAAATATACGCCGAC | TATAATGGAGGTAAAGATTG | TGTTCCGGTCTTACCTGCAG |
| **101530 Δ/Δ_A/B** | gtcagcggccgcatccctgcAGAGGTGGGGGGGGGGGGTT | GGAGCTCGAGCTAAAGGTTT | GTCAACACAACTCAAAAGAA | TACAATCACGGATATAATCA | ATGTGGTATCGTCGTGGTGG |
| **802440 Δ/Δ_A/B** | gtcagcggccgcatccctgcAGAGTGAGCCAAAGGTTGGG | GTTGCGATGTGGAGGTAGGT | GAAGTAACCGTAGCCTTTCC | GTGATTATGTACAAGTTTGA | TAGGAGGACCCGATTGCTCA |
| **203540 Δ/Δ_A/B** | gtcagcggccgcatccctgcGAGGTGTTACACCACGAAAG | GAAGAATCACCAAAATTGAT | TAACGGACAATTAATGTCTT | AAAACGGCAAACATTTCGGG | CTGTGCTTGTTGTGGTGGTG |
| **204950 Δ/Δ_A/B** | gtcagcggccgcatccctgcCTATACTACTTTAGATGCCC | AAAGTCAATCGTCGTCAGTT | TAACATAGCCCGGTATAAAT | CATTCTTACCCAGTTGGAAG | TCCTTGACCAATTGCTCCGT |
| **302310 Δ/Δ_A/B** | gtcagcggccgcatccctgcGCGGTGCTTGGGTGTTTCAT | GTGGCAATCACCTTCGATTG | ACGAGTATCTTGAAAATTTG | TTGTTCGTCTTTGGTCAATG | TTTCCGCGTCATGTCCTTGA |
| **802880 Δ/Δ_A/B** | gtcagcggccgcatccctgcATTGAAATTTTGTTTGTATT | GACAAATTTGGTTGTGGCAT | TCACGTGGTCCAACTTGATT | TTCAATTGGACATTCTCACT | TTTCCTTCCACGCCTCTTCC |
| **302230 Δ/Δ_A/B** | gtcagcggccgcatccctgcCCGCTTTCGAACCTGTTTTG | TAGATTTACCAGGACTTCTA | TGGGTGATTGTGTAAGCCTT | GCAACTGGGGTTGTGGTTGT | TTACACATCCGATGGGGCAC |
